# Development and Interfacial Mechanism of Epoxy Soybean Oil-Based Semi-Liquid Gel Materials for Wellbore Sealing Applications

**DOI:** 10.3390/gels11070482

**Published:** 2025-06-22

**Authors:** Yuexin Tian, Yintao Liu, Haifeng Dong, Xiangjun Liu, Jinjun Huang

**Affiliations:** 1 Petroleum Engineering Technology Institute of Southwest Petroleum Branch, China Petroleum & Chemical Corporation (SINOPEC), Deyang 618000, China; liuyintao.xnyq@sinopec.com (Y.L.); donghaifeng.xnyq@sinopec.com (H.D.); 2 State Key Laboratory of Oil and Gas Reservoir Geology and Exploitation, Southwest Petroleum University, Chengdu 610500, China; 13880093092@163.com (X.L.); huangjjswpu@163.com (J.H.)

**Keywords:** semi-liquid gel, epoxy soybean oil, crosslinked network, wellbore sealing, interfacial adhesion, degradable gel, smart sealing material

## Abstract

In this study, a novel semi-liquid gel material based on bisphenol A-type epoxy resin (E51), methylhexahydrophthalic anhydride (MHHPA), and epoxidized soybean oil (ESO) was developed for high-performance wellbore sealing. The gel system exhibits tunable gelation times ranging from 1 to 10 h (±0.5 h) and maintains a low viscosity of <100 ± 2 mPa·s at 25 °C, enabling efficient injection into the wellbore. The optimized formulation achieved a compressive strength exceeding 112.5 ± 3.1 MPa and a breakthrough pressure gradient of over 50 ± 2.8 MPa/m with only 0.9 PV dosage. Fourier transform infrared spectroscopy (FTIR) confirmed the formation of a dense, crosslinked polyester network. Interfacial adhesion was significantly enhanced by the incorporation of 0.25 wt% octadecyltrichlorosilane (OTS), yielding an adhesion layer thickness of 391.6 ± 12.7 nm—approximately 9.89 times higher than that of the unmodified system. Complete degradation was achieved within 48 ± 2 h at 120 °C using a γ-valerolactone and p-toluenesulfonic acid solution. These results demonstrate the material’s potential as a high-strength, injectable, and degradable sealing solution for complex subsurface environments.

## 1. Introduction

Liquid rubber plug plugging technology (TRPPT) involves the injection of a reactive liquid rubber solution into the bottom of a wellbore through coiled tubing, where it undergoes in situ curing to form a semi-solid or gel-like plug under reservoir conditions [1,2]. This cured plug serves to effectively seal the wellbore, providing stable pressure conditions for subsequent string operations [3,4,5]. After operations are completed, the cured polymer can be degraded by introducing a chemical breaker [6]. With the ongoing development of deep and ultra-deep gas reservoirs, the bottomhole environment is becoming increasingly harsh in terms of temperature and pressure [7]. Therefore, it is necessary to enhance the performance of TRPPT materials to ensure operational safety and pressure control in challenging formations such as gas storage wells, reinjection wells, and leakage-prone wells.

Conventional plugging materials used in oilfields include polymer-based agents [8], solid-phase particulates [9], fibers [10], expandable particles [11], cementitious plugs [12], foams [13], and vesicle fluid systems [14]. However, many of these suffer from significant limitations: particulate- and fiber-based agents often fail to bridge large boreholes [15], cement plugs lack degradability and retrievability [16], and vesicle-based fluids are complex to prepare and unsuitable for high-temperature applications [15]. Foam-based agents exhibit short plugging durations and insufficient strength [17]. In contrast, liquid rubber plug materials composed of polymeric gels and resins offer improved conformance and adaptability due to their injectable nature and in situ solidification [18]. However, polymer gels still face challenges in mechanical robustness [19] and interfacial adhesion to rock surfaces [20]. Additionally, their gelation behavior can be difficult to control precisely, and large dosages are often needed to achieve effective sealing.

Epoxy resin systems have emerged as promising alternatives due to their excellent mechanical properties and inherent degradability. Leng studied the effects of diluent content on the injectability of epoxy-based plugging agents [20], while Chen developed an epoxy resin formulation for sealing fractures [21]. Nevertheless, commercial epoxy resins present several challenges, including mismatched gelation times, high initial viscosity, and limited degradability once cured [22]. In comparison, polyester-type resins show rapid degradation under thermal or chemical conditions [23]. Among the available degradation pathways for epoxy systems—mechanical [24], thermal [25], and chemical [26]—chemical degradation remains the most effective for downhole applications. For example, degradation in supercritical environments such as supercritical water enables rapid network breakdown [27,28]. Zhang demonstrated complete degradation of epoxy/anhydride systems using a mixture of dodecylbenzenesulfonic acid (DBSA) and water at 170 °C [28]. Yang proposed polyethylene glycol/sodium hydroxide mixtures [29], and more recently, Zhang introduced a green solvent system based on γ-valerolactone (GVL)-H_2_O and p-toluenesulfonic acid for fast, mild degradation of epoxy resins [30].

Comparative analysis with recent epoxy and bio-based sealing systems further confirms the superiority of the present design. Gao et al. [31] and Wu et al. [32] achieved high strength and acid-triggered degradation, yet neither addressed flowability or interfacial bonding—critical properties for wellbore sealing. Meanwhile, Liu et al. [33] developed a thermally reversible bio-based sealing material with an elongation at break exceeding 400%, while Zhang et al. [34] constructed a modified starch-based grout exhibiting good water tightness. However, both materials exhibit compressive strengths below 1.5 MPa and service temperatures below 100 °C, limiting their applicability in high-temperature and high-pressure wellbore sealing environments.

In this study, a high-strength, biodegradable gel material was developed by combining epoxy resin (E51) with methylhexahydrophthalic anhydride (MHHPA), further modified with n-butyl glycidyl ether, epoxidized soybean oil (ESO), and octadecyltrichlorosilane (OTS). The resulting semi-liquid gel network exhibited tunable curing (1–10 h), excellent injectability (<100 mPa·s), and strong interfacial adhesion (bonding layer ~391.6 nm), making it suitable for complex downhole sealing.

Compared with recent degradable epoxy or bio-based sealants, which often suffer from low strength or poor interfacial bonding, the present formulation demonstrates superior compressive strength (>112 MPa) and rapid thermal degradation (within 48 h at 120 °C). These combined properties highlight its potential as a robust, degradable temporary plugging material for harsh subsurface environments.

## 2. Results and Discussion

### 2.1. Preparation and Characterization of Liquid Rubber Plug

**Figure 1:**
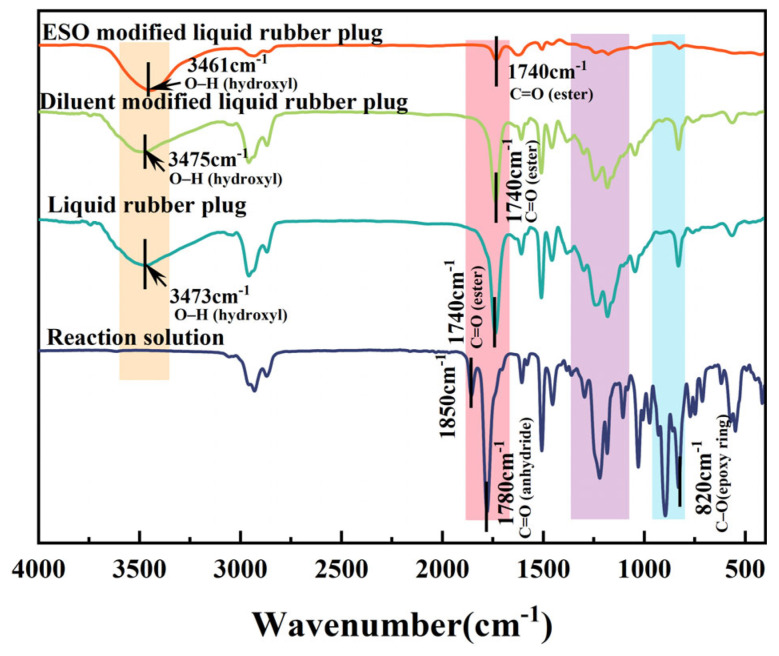
FTIR spectra of the reaction solution and cured liquid rubber plugs with and without diluent and ESO modification. The disappearance of absorption peaks at 1850 cm^−1^ and 1780 cm^−1^ (C=O stretching of anhydride) and at 820 cm^−1^ (epoxy ring) confirms the completion of the epoxy–anhydride crosslinking reaction. The emergence of a new ester peak at 1740 cm^−1^ and broad O–H stretching bands in the range of 3460–3475 cm^−1^ indicates the successful ring-opening of epoxidized soybean oil and formation of a polyester network structure.

The liquid rubber plug was prepared using E51 epoxy resin, MHHPA, and N, N-dimethylbenzylamine and was modified by n-butyl glycidyl ether and ESO to improve the rheology and compressive strength. As shown in Figure 1, the epoxy group of the epoxy resin reacts with the anhydride group, as confirmed by the disappearance of the stretching vibration peaks of the anhydride group at 1780 cm^−1^ and 1850 cm^−1^ and the epoxy group at 820 cm^−1^ in the reaction solution, and the formation of a large number of ester bonds at 1740 cm^−1^ and hydroxyl peaks around 3460–3475 cm^−1^. The infrared spectrogram of the liquid rubber plug was essentially the same as that of the diluent-modified liquid rubber plug, without the generation of new groups, indicating that the introduction of n-butyl glycidyl ether had a minor effect on the reaction mechanism. The intensity of the characteristic hydroxyl peaks increased dramatically after the addition of ESO, which was attributed to the formation of a large number of hydroxyl groups after the ring-opening of the epoxidized soybean oil. In conclusion, liquid rubber plugs constructed with ESO and E51 modified epoxy resin/anhydride are polyester structures.

**Figure 2:**
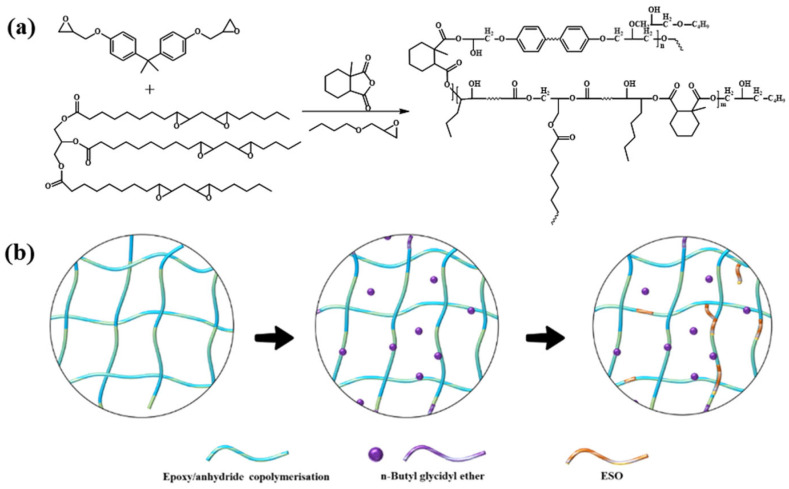
Schematic illustration of the curing and reinforcement mechanism of the liquid rubber plug system: (**a**) ring-opening copolymerization reaction between epoxy resin (E51), methylhexahydrophthalic anhydride (MHHPA, acting as both curing agent and crosslinker), epoxidized soybean oil (ESO), and n-butyl glycidyl ether; (**b**) conceptual diagram of the resulting ester-crosslinked polymer network and enhanced interfacial bonding.

The ester-bond-crosslinked polymer has flexibility and polarity. It increases the interactions between molecular chains, endows the material with flexibility, and improves the toughness of the material. The hydroxyl group is a strongly polar group that can form hydrogen bonds with surrounding molecules (Figure 2). The E51 epoxy resin used in this study has an average molecular weight of approximately 380 g/mol and an epoxy equivalent weight of ~190 g/eq, corresponding to a diglycidyl ether of bisphenol-A (DGEBA) backbone with bifunctional reactive sites. In this epoxy–anhydride system, methylhexahydrophthalic anhydride (MHHPA) serves dually as a curing agent and a crosslinker, participating in ring-opening reactions with multiple epoxy groups to form a densely crosslinked polyester network. This structural framework forms the backbone of the gel matrix and supports further reinforcement by diluents and ESO. Their effects on mechanical strength and interfacial performance are further analyzed in Section 2.3 and Section 2.5.

To further characterize the physical properties of the uncured gel system, particle size distribution analysis was conducted using a laser diffraction particle size analyzer at 25 °C. The formulation was diluted with isopropanol and sonicated to ensure uniform dispersion prior to testing.

**Figure 3:**
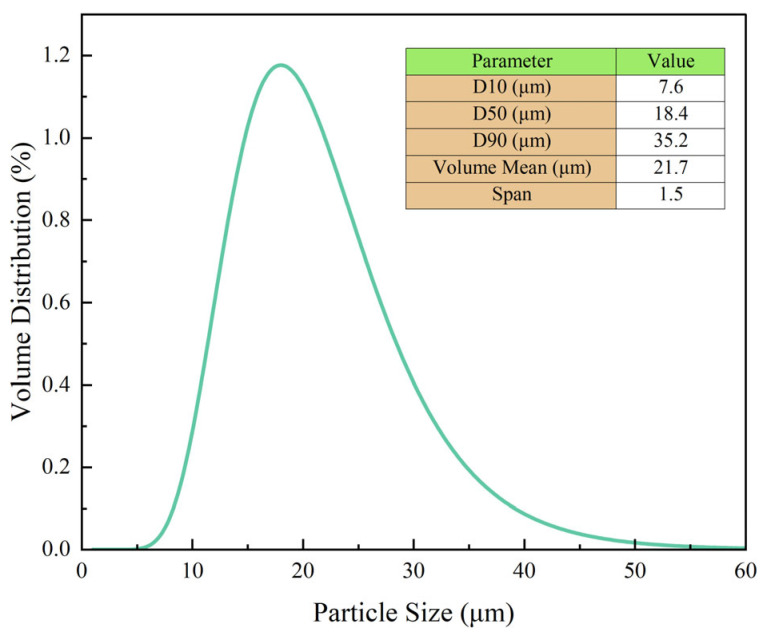
Particle size distribution of the uncured liquid rubber plug precursor, indicating uniform dispersion and optimal filler distribution for enhanced sealing properties.

As shown in Figure 3, the volume-based average particle size of the gel formulation was 21.7 μm, with D10, D50, and D90 values of 7.6 μm, 18.4 μm, and 35.2 μm, respectively. The calculated span was 1.5, indicating a relatively narrow size distribution. No signs of bimodal distribution or significant agglomeration were observed, which confirms a homogeneous dispersion state. Such uniformity contributes to excellent fluidity before gelation and ensures consistent injectability and crosslinking performance in the wellbore.

### 2.2. Rheology

**Figure 4:**
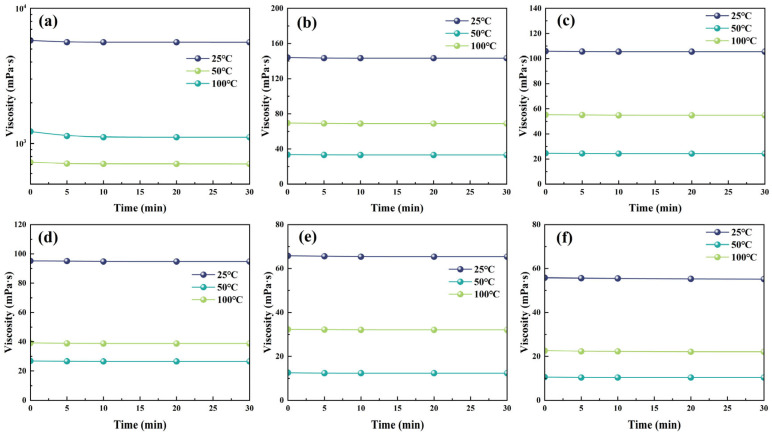
Time-dependent viscosity profiles of liquid rubber plug systems with different diluent dosages at 25 °C, 50 °C, and 100 °C. Subfigures (**a**–**f**) correspond to decreasing diluent dosages from 0 wt% to 16 wt%. The initial formulation without diluent (**a**) exhibits extremely high viscosity (>5000 mPa·s at 25 °C), while increasing diluent content significantly reduces viscosity across all temperatures, enhancing injectability.

**Figure 5:**
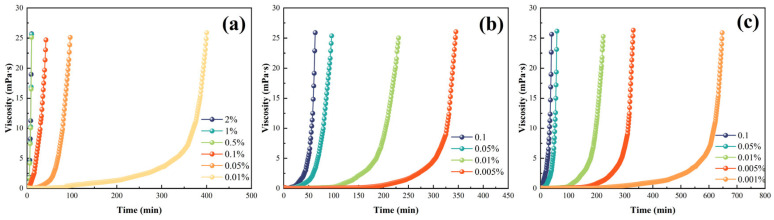
Viscosity–time curves of liquid rubber plug systems at different temperatures and initiator dosages: (**a**) 120 °C, (**b**) 130 °C, and (**c**) 140 °C. All curves exhibit a characteristic sharp rise in viscosity indicative of epoxy–anhydride gelation onset.

The effects of diluent and initiator dosages on the rheological properties of liquid rubber plugs were investigated to validate the injection properties of the liquid plug reaction solutions in wellbores. Figure 4 shows the apparent viscosities at 25, 50, and 100 °C with different diluent dosages. Reactive solutions without diluents are difficult to inject into the wellbore because of their high apparent viscosity, which is greater than 5000 mPa·s at 25 °C and remains at 705 mPa·s even at 100 °C. The apparent viscosity decreased dramatically after the addition of the diluent. The apparent viscosity was lower than 100 mPa·s at 25 °C and 25 mPa·s at 100 °C when the diluent dosage was 12~16 wt%. Figure 4 shows the effects of initiator dosage on the apparent viscosity of the reactive solutions at 120, 130, and 140 °C. The viscosity–time curves in Figure 5a–c all showed a right-angle rise, which is in line with the right-angle curing theorem of epoxy resin. In addition, the time of the low-viscosity area (the viscosity is lower 100 mPa·s) can be regulated by changing the initiator dosage in the liquid plug reaction solution; for example, the initiator dosage was in the range of 0.01–0.05 wt% at 120 °C, 0.005–0.05 wt% at 130 °C, and 0.001–0.01 wt% at 140 °C, which provides a guarantee for mixing and injection of the base fluid at 25 °C.

**Figure 6:**
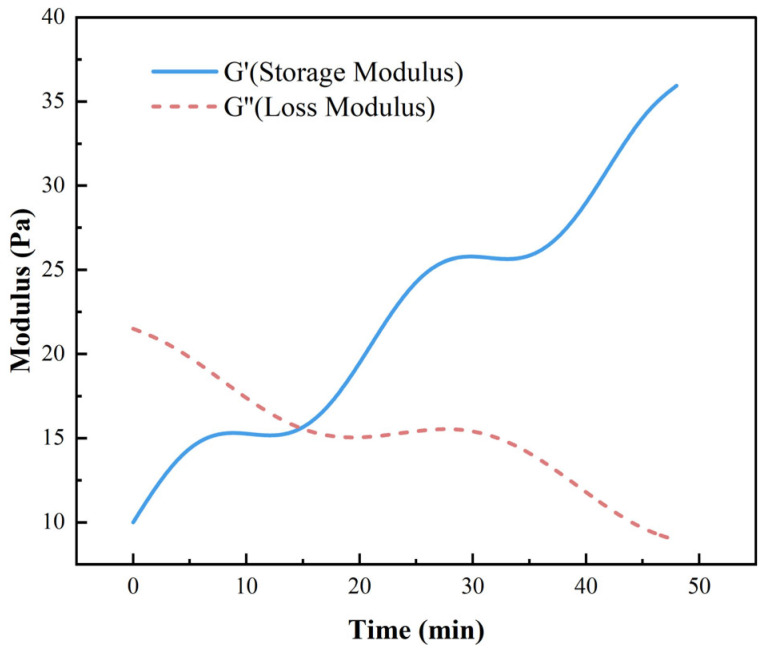
Time-dependent evolution of viscoelastic moduli during gelation of the liquid rubber plug system at 120 °C.

In addition to steady-state viscosity measurements, oscillatory rheological analysis was conducted to assess the viscoelastic evolution of the gel system during curing. As shown in Figure 6, the storage modulus (G′) and loss modulus (G″) were recorded over time at 120 °C. Initially, G″ > G′ indicates a viscous-dominated system. As the crosslinking progressed, both G′ and G″ increased, with G′ surpassing G″ at around 24 min, marking the sol–gel transition point. G′ continued to rise steadily thereafter, reaching 36.3 Pa at 45 min, reflecting the formation of a robust crosslinked elastic network. This dynamic viscoelastic behavior complements the compressive strength data discussed in Section 2.3 and confirms the feasibility of in situ gelation under high-temperature wellbore conditions.

### 2.3. Compressive Strength Regulation Mechanism

**Figure 7:**
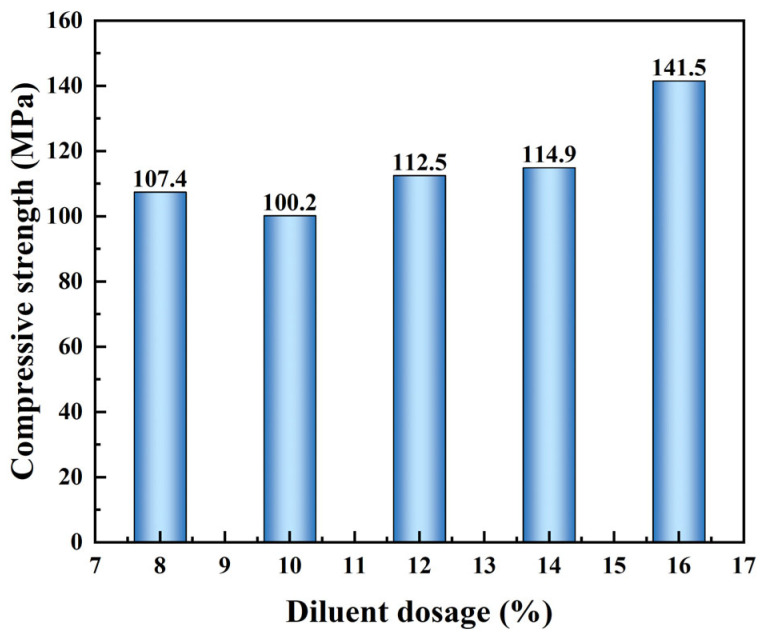
Effect of diluent dosage on the compressive strength of cured liquid rubber plugs. The strength initially fluctuates and then increases significantly as the dosage rises from 8% to 16 wt%, reaching a peak of 141.5 MPa.

To investigate the influence of diluent on the mechanical performance of liquid rubber plugs, the effect of n-butyl glycidyl ether dosage on compressive strength was examined (Figure 7, Table 1). As the diluent content increased from 8 wt% to 16 wt%, the compressive strength of the cured plugs increased from 101.2 MPa to a peak of 141.5 MPa. This trend suggests that the diluent participated in the epoxy/anhydride cross-linking reaction, reducing network rigidity while enhancing the material’s elasticity. The presence of reactive diluents improves the flexibility of polymer chains and supports energy dissipation under compression, thus increasing overall strength.

Moreover, the addition of diluents reduces the viscosity of the reaction mixture, which facilitates better interfacial contact and mold filling during curing. This promotes uniform network formation and enhances long-term sealing performance under complex reservoir conditions. However, excessive diluent loading (beyond 16 wt%) may reduce cross-linking density to a point where gel curing is incomplete or compromised, leading to poor plug integrity.

From a molecular perspective, the introduction of a diluent decreases the cross-linking density between epoxy chains, thereby weakening intermolecular forces and increasing chain flexibility [35,36]. This enhanced flexibility allows the polymer network to undergo greater elastic deformation before fracture, improving material toughness. Additionally, moderate diluent addition promotes homogeneous mixing within the resin system, mitigating stress concentrations and lowering the risk of brittle fracture initiation points. These effects collectively enhance the compressive strength and failure resistance of the cured plugs. Nevertheless, excessive diluent disrupts the curing balance and leads to an under-crosslinked structure, compromising plug effectiveness.

In addition to diluent modification, epoxy soybean oil (ESO) was introduced as a reactive toughening agent. Owing to its epoxy functional groups, ESO participates in the epoxy–anhydride cross-linking reaction, forming a polyester-like copolymer network with enhanced chain flexibility. This reactive incorporation avoids phase separation and improves network homogeneity compared to physical blending methods.

**Figure 8:**
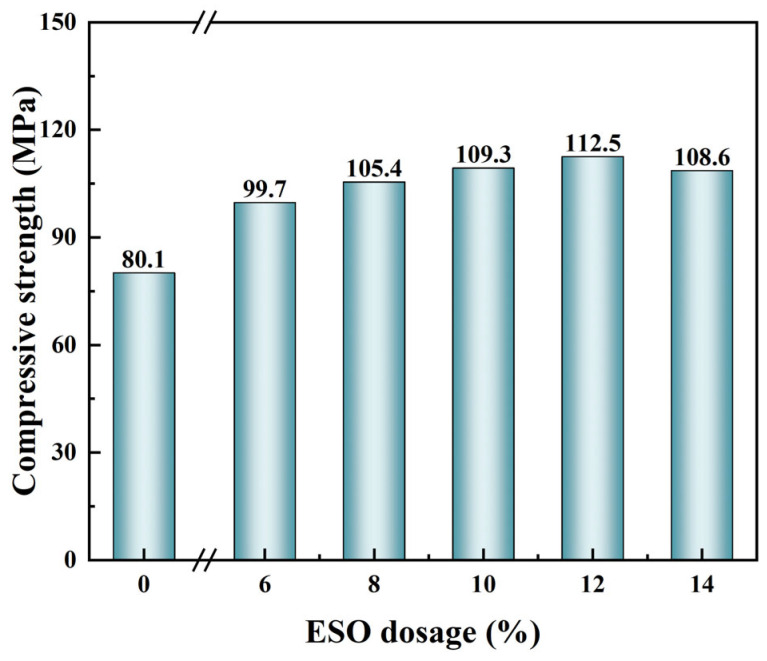
Compressive strength of cured liquid rubber plugs as a function of ESO dosage. The strength increases steadily from 80.1 MPa (0%) to a maximum of 112.5 MPa at 12 wt%, then slightly declines at 14 wt%.

**Figure 9:**
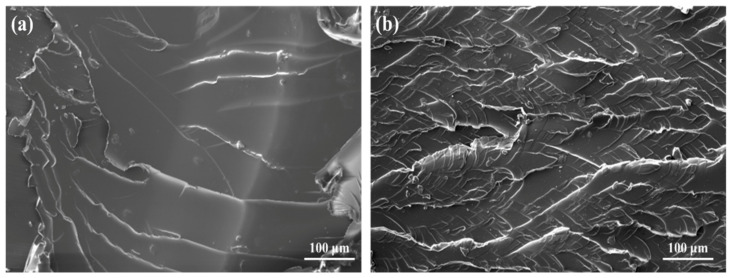
SEM images showing the fracture surface morphology of liquid rubber plugs: (**a**) unmodified plug with smooth, brittle fracture planes; (**b**) ESO-modified plug displaying rougher surfaces and pronounced crazing, indicative of enhanced ductility and energy dissipation capacity under compressive loading.

As shown in Figure 8, increasing ESO dosage enhanced the compressive strength up to a maximum of 112.5 MPa at 12 wt%. Beyond this point, further addition of ESO (14 wt%) led to a slight decline in strength due to excessive flexibility and reduced structural rigidity. Figure 9a,b presents SEM images of the fracture surfaces before and after modification. The unmodified plug displayed a smooth and brittle fracture plane (Figure 9a), while the ESO-modified sample showed rough surfaces and visible crazing features (Figure 9b), indicating a transition from brittle to ductile failure. These observations confirm that ESO promotes plastic deformation and energy dissipation, reinforcing the compressive and sealing capabilities of the plugs.

### 2.4. Curing Time Regulation Mechanism

To initiate the ring-opening polymerization of epoxy and anhydride monomers, a tertiary amine catalyst was required, as these groups exhibit negligible reactivity under thermal conditions alone within 120–140 °C. In this study, N,N-dimethylbenzylamine was selected as the initiator, which effectively triggered the epoxy–anhydride reaction through catalytic activation. However, excessive initiator loading led to uncontrollably rapid gelation and poor curing manageability. Meanwhile, temperature played a synergistic role with the initiator concentration by accelerating the molecular mobility and lowering the energy barrier for the ring-opening reaction.

**Figure 10:**
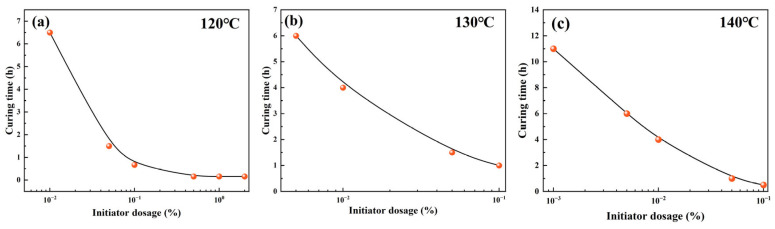
Curing time of liquid rubber plugs as a function of initiator dosage under different temperatures: (**a**) 120 °C, (**b**) 130 °C, and (**c**) 140 °C. Increasing initiator dosage leads to exponential reduction in curing time, with faster gelation at higher temperatures (Note: The number of data points varies among subfigures due to rapid gelation under elevated temperatures and initiator dosages).

Therefore, a parametric investigation was conducted to assess the influence of both initiator dosage (0.001–0.1 wt%) and curing temperature (120–140 °C) on the curing time of the liquid rubber plug system. As illustrated in Figure 10a–c, both increasing initiator concentration and temperature resulted in exponential reductions in curing time. At 140 °C with a 0.1 wt% initiator, complete gelation occurred within 0.5 h, whereas at 120 °C with a 0.01 wt% initiator, the system required over 4 h to cure. These results indicate that the curing time can be flexibly tuned in the range of 0.5–5 h by jointly modulating the initiator content and temperature, offering valuable adaptability for downhole operation windows.

All experiments were performed in triplicate, and the curing time values were reported with a standard deviation of ±0.5 h, ensuring the reliability and reproducibility of the measurements.

### 2.5. Interfacial Adhesive Performance and Mechanism

**Figure 11:**
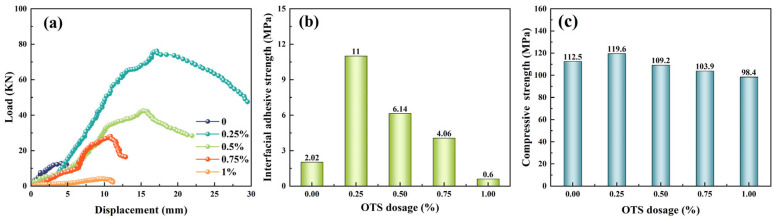
Effect of OTS dosage on interfacial and mechanical performance of liquid rubber plugs: (**a**) Load–displacement curves at various OTS contents, (**b**) corresponding interfacial adhesive strengths, and (**c**) bulk compressive strengths.

Different amounts of OTS were added to modify the interfacial adhesive strength of liquid rubber plugs, as shown in Figure 11. The adhesive strength and compressive strength both showed an initial increase followed by a decrease with increasing OTS content. At a dosage of 0.25 wt%, the compressive strength reached 119.6 MPa (a 6.3% increase), while the interfacial adhesive strength increased by 445%. According to Table 2, the plugs formed a robust polymer layer at the tubing interface when the OTS dosage ranged from 0 to 0.5 wt%. This improvement is attributed to the amphiphilic nature of OTS, which reacts preferentially with hydroxyl groups on the tube wall to create a strong adhesive bond. However, excessive OTS disrupts the polymer network structure, reducing both adhesive and mechanical properties.

**Figure 12:**
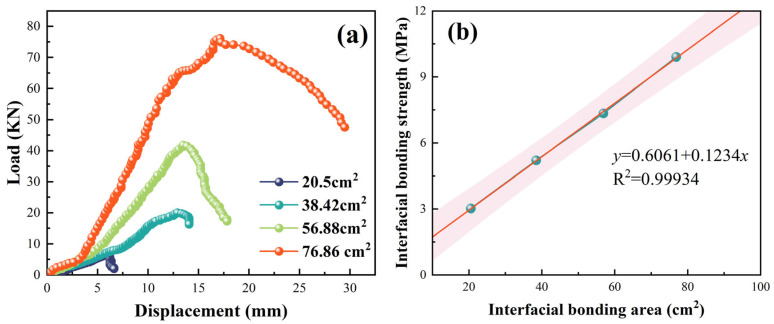
Effect of interfacial bonding area on mechanical adhesion performance of liquid rubber plugs: (**a**) load–displacement curves under different bonded areas (20.5 to 76.86 cm^2^); (**b**) fitted relationship between bonding area and interfacial bonding strength, showing a strong linear correlation (R^2^ = 0.99934).

The effect of the interfacial adhesive area on the interfacial adhesive strength was investigated via roll-out experiments, as shown in Figure 12. In a simulated wellbore with an inner diameter of 40 cm, the strength was 3.02 MPa at an interfacial adhesive area of 15.7 cm^2^ and 9.91 MPa at an interfacial adhesive area of 62.8 cm^2^, and the interfacial adhesive strength increases with the increase in adhesive area (Figure 12a). Figure 12b shows the data fitting. The interfacial adhesive area and adhesive strength had a linear relationship: *y* = 0.512 + 0.121*x*. Increasing the interfacial adhesive area can increase the strength of the adhesive.

**Figure 13:**
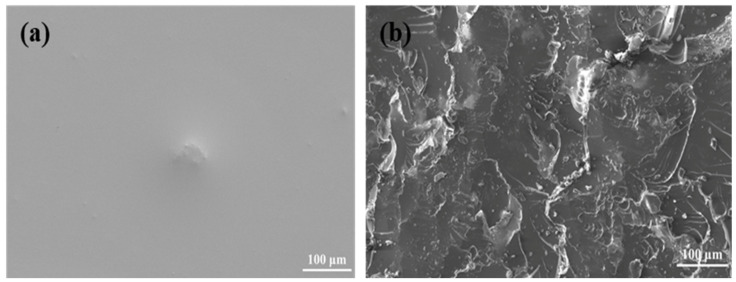
SEM images of the adhesive interfacial micromorphology between liquid rubber plugs and steel tubing: (**a**) unmodified plug (0 wt% OTS) shows a smooth, featureless interface indicative of poor bonding; (**b**) plug modified with 0.25 wt% OTS displays a roughened, continuous adhesive layer with embedded microtextures, suggesting enhanced chemical and physical interfacial interactions.

**Figure 14:**
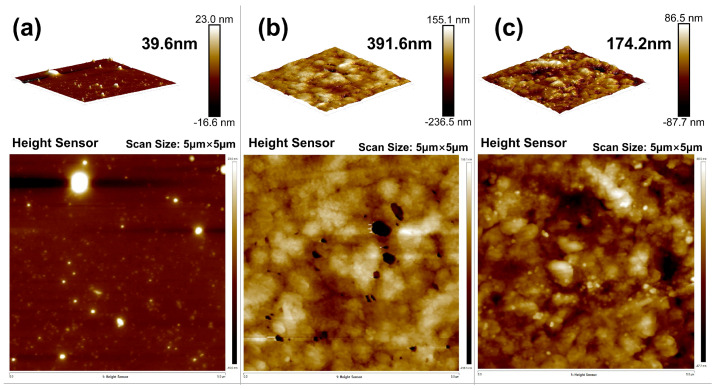
Atomic force microscopy (AFM) height profiles of the adhesive interfacial layer under different OTS dosages: (**a**) 0 wt% OTS—flat and poorly bonded surface with a maximum adhesion thickness of 39.6 nm; (**b**) 0.25 wt% OTS—continuous interfacial embedding with a maximum adhesion thickness of 391.6 nm, indicating enhanced bonding performance; (**c**) 0.5 wt% OTS—reduced interfacial continuity with adhesion thickness of 174.2 nm.

Figure 13a shows the microstructure of the surface layer of the unmodified liquid glueplugs, which was uniform and did not exhibit any obvious stress damage during push-out. Atomic force microscopy (AFM) (Figure 14a) revealed that the thickness of the adhesive in the surface layer of the liquid rubber plug was small, with a maximum gap of 39.6 nm. Figure 13b shows the microstructure of the surface layer of the 0.25% wtOTS-modified liquid rubber plugs. Traces of damage to the surface layer of the liquid rubber plugs can be clearly observed. Figure 14b shows that the adhesion thickness of its surface layer reaches 391.6 ± 12.7 nm, calculated from multiple AFM line scans, which is improved by 9.89 times compared with the unmodified liquid rubber plugs. The compressive strength of the liquid rubber plugs modified with 0.5 wt% OTS dosage was reduced, as was the interfacial adhesive strength, as indicated by the AFM (Figure 14c), which showed that the adhesive thickness was 174.2 nm. It was shown that OTS effectively modified the adhesive properties between the liquid rubber plug and pipe wall.

However, it is important to emphasize that scanning electron microscopy (SEM) provides only qualitative evidence of surface continuity and cannot directly quantify interfacial bonding strength. Therefore, the conclusion regarding enhanced adhesion is primarily supported by mechanical testing using a Universal Testing Machine (UTM). As shown in Figure 11b, the interfacial adhesive strength increased significantly with the addition of OTS, peaking at 11.0 MPa under 0.25 wt% dosage—representing a 445% increase compared to the unmodified sample (2.02 MPa). At higher dosages (≥0.5 wt%), the strength gradually declined due to phase separation and possible over-saturation of silane at the interface.

Moreover, the enhancement trend observed in UTM tests is in good agreement with the improved interfacial microstructure revealed by SEM, suggesting that OTS modification improves both morphological embedding and mechanical bonding at the interface. The enhanced interfacial adhesion is attributed to the dual mechanism of chemical bonding between OTS and hydroxyl-rich tubing surfaces and physical interaction between the hydrophobic alkyl chains and the polymer matrix. This synergy establishes a stable adhesive interface. However, excessive OTS disrupts the polymer network and weakens both interfacial adhesion and compressive strength.

To further substantiate the interfacial performance and guide future gel plug design, we reviewed recent advancements in intelligent gel systems and their response mechanisms under complex loading conditions. He et al. [37] introduced peptide-based hydrogels with reversible supramolecular interactions, enabling dynamic and programmable adhesion. While our system does not yet exhibit reconfigurability, the enhanced interfacial adhesion induced by OTS modification parallels the principle of molecular anchoring. On the other hand, Fang et al. [38] emphasized the role of multiaxial thermal–mechanical stress in controlling deformation and sealing behavior. Though our current study focuses on static loading, future work may incorporate stress-coupled evaluations. Furthermore, the plug integrity analysis under thermal-induced wellhead growth [39] points to the necessity of considering lifecycle-induced interface fatigue and long-term thermal degradation. Collectively, these studies suggest that integrating molecular interfacial design with environmental adaptability and fatigue modeling may enhance the functional robustness of semi-liquid gel systems. Our epoxy–ESO-based formulation presents a foundational step toward such integrative designs.

### 2.6. Plugging Property and Degradation Performance

**Figure 15:**
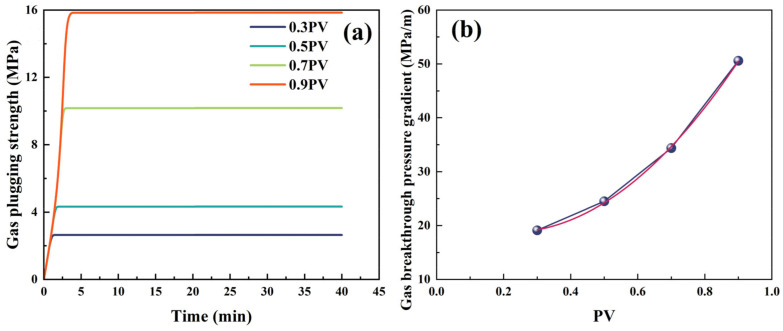
Gas plugging performance of liquid rubber plugs under constant loading conditions. (**a**) Time-dependent gas plugging strength at various injection volumes (0.3–0.9 PV); higher volumes result in greater pressure retention capacity. (**b**) Fitted relationship between injected volume (PV) and breakthrough pressure gradient, showing an exponential increase, reaching over 50 MPa/m at 0.9 PV. The purple dots represent experimental data, and the red curve indicates the fitted exponential trend.

**Figure 16:**
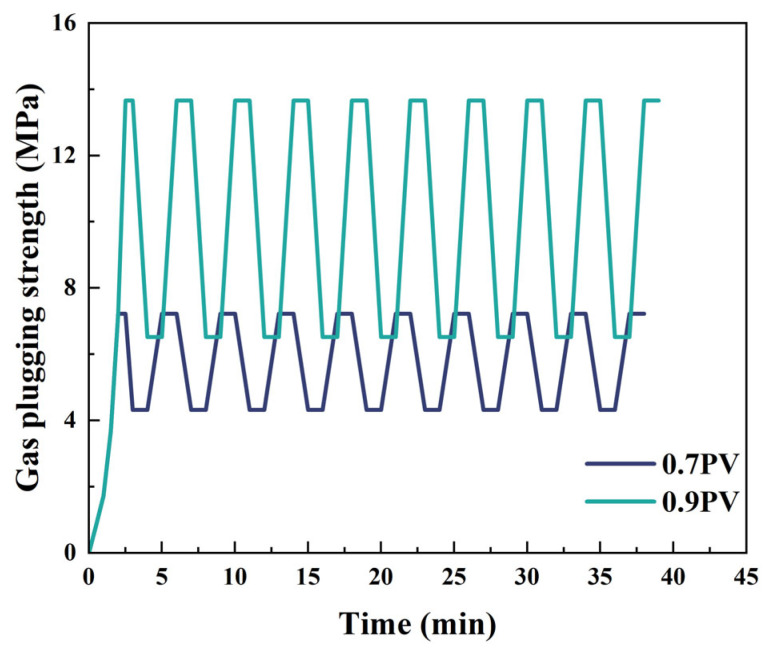
Plugging performance of liquid rubber plugs under alternating gas loads.

The gas plugging performance of different liquid rubber plug injection volumes was investigated using simulated plugging experiments. Figure 15 and Figure 16 show the plugging performance of the liquid rubber plug under constant and alternating gas loads, respectively. The liquid rubber plug dosage increased exponentially with the gas breakthrough pressure gradient. Figure 15a shows that the plugging strengths were 2.64 MPa, 4.32 MPa, 10.17 MPa and 15.84 MPa after injecting with 0.3, 0.5, 0.7 and 0.9 PV (where PV refers to the total pore volume of the core sample) of the liquid rubber plug reaction solution, respectively. Figure 15b shows the fitted relationship between the interfacial adhesive area and gas breakthrough pressure gradient, which grows exponentially, *y* = 21.81 − 0.123*x* + 0.00122*x*^2^. This indicates that as the interfacial adhesive area increased, both the gas containment strength and the breakthrough pressure gradient increased. Meanwhile, the breakthrough pressure gradients of the gel system increased with the injection volume, as follows: 19.11 MPa/m at 0.3 PV, 24.50 MPa/m at 0.5 PV, 34.38 MPa/m at 0.7 PV, and 50.59 MPa/m at 0.9 PV. Based on these values and the method described in Section 4.2.7, the effective permeability after plugging was calculated using Darcy’s law. The permeability reduction percentage was calculated by comparing the post-plugging permeability with the initial permeability of the sand-filled tube (132.6 mD). The detailed results are presented in Table 3, showing that the permeability decreased significantly with increasing plug volume, indicating effective gas sealing performance.

These results indicate a clear inverse relationship between the injected plug volume and the post-plugging permeability. At only a 0.3 PV dosage, the permeability was reduced by 85.6%, and further decreased to 61.9% at 0.9 PV. Such significant reductions, derived from the Darcy-based permeability estimations in Section 4.2.7, demonstrate the system’s capacity to form dense and robust plugging structures even at low dosages, highlighting its practical advantage in minimizing material usage while maximizing sealing effectiveness.

Figure 16 shows that under 10 times of alternating loads, the plugging strength of the liquid rubber plug is 13.66 MPa and 7.22 MPa, respectively, which indicates that the liquid rubber plug can achieve stable plugging under the complex force.

These results confirm that effective sealing performance can be achieved with minimal gel volume through optimized injection.

The figure shows the response of gel plugs (0.7 PV and 0.9 PV) to cyclic loading over 10 cycles at 120 °C.

**Figure 17:**
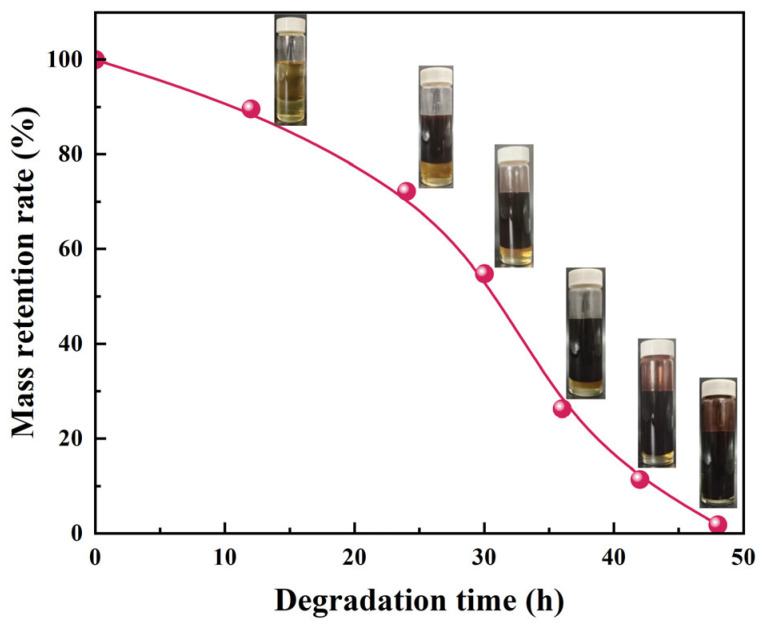
Degradation performance of liquid rubber plug in γ-valerolactone/p-toluenesulfonic acid solution at 120 °C.

Figure 17 shows the effect of compounding p-toluenesulfonic acid and γ-valerolactone on the degradation behavior of the liquid rubber plug. Under single-sided contact at 120 °C and an acid-to-solvent mass ratio of 1:4, the plug was fully degraded to a fluid state within 48 h. The curve displays a continuous decrease in mass, indicating sustained degradation over time. Inset images reveal the morphological evolution of the gel sample: it remains intact at 0 h, gradually darkens and softens by 20–30 h, and becomes fully liquefied after 48 h. This visually confirms the effective penetration and breakdown of the polymer network in the acidic degradation medium.

Although the gel material demonstrated excellent short-term sealing capability and complete degradation within 48 h at 120 °C, its long-term stability under dynamic downhole conditions such as sustained pressure, thermal fluctuation, and chemical exposure remains to be fully validated. These aspects are currently under investigation and will be addressed in future studies to ensure the reliability of the material in extended sealing operations.

The mass retention rate decreases progressively over 48 h, indicating continuous polymer breakdown.

## 3. Conclusions

This study developed a degradable semi-liquid rubber plug system featuring adjustable mechanical properties, controllable curing behavior, robust interfacial adhesion, and environmentally responsive degradation. Through the synergistic incorporation of reactive diluents and epoxy soybean oil-based toughening agents, the material exhibited enhanced compressive strength and elastic deformation capacity. The OTS-modified surface improved the bonding strength with formation interfaces, ensuring stable sealing under both static and alternating gas loads. Moreover, the plug demonstrated effective degradation in γ-valerolactone/p-toluenesulfonic acid at 120 °C within 48 h, indicating its feasibility for temporary zonal isolation.

In addition to performance characterization, this work elucidated the underlying mechanisms governing strength regulation, curing kinetics, interfacial reinforcement, and degradation behavior. These findings collectively contribute to a comprehensive design strategy for advanced degradable plug systems capable of adapting to complex downhole environments. The insights gained provide a foundation for future optimization toward intelligent, self-adaptive sealing materials.

## 4. Materials and Methods

### 4.1. Materials

Epoxy resin (E51, purity 98%, average molecular weight ~380 g/mol, epoxy equivalent weight ~190 g/eq, functionality ≈ 2), n-butyl glycidyl ether (purity 98%), methylhexahydrophthalic anhydride (purity 98%, MHHPA), octadecyltrichlorosilane (purity 98%, OTS), gamma-valerolactone (purity 98%), p-toluenesulfonic acid (purity 98%), epoxied soybean oil (purity 98%, ESO), γ-Valerolactone (purity 98%), p-toluenesulfonic acid (purity 98%), N, N-dimethylbenzylamine (purity 98%) and methylhexahydrophthalic anhydride (MHHPA, 98%) were purchased from the Sinopharm Group Chemical Reagent Co. Ltd., Chengdu, China. All chemicals were used without further purification.

### 4.2. Experimental Methods

#### 4.2.1. Preparation of Liquid Rubber Plug

The liquid rubber plug formulation was prepared by mixing 3.92 g of epoxy resin (E51), 3.53 g of methylhexahydrophthalic anhydride (MHHPA), 1.37 g of n-butyl glycidyl ether, 1.18 g of epoxidized soybean oil (ESO), and 0.025 g of octadecyltrichlorosilane (OTS) in a 50 mL beaker. To initiate the curing process, 0.001–0.1 wt% of N,N-dimethylbenzylamine (BDMA) was added as a tertiary amine catalyst. The mixture was stirred thoroughly at room temperature (25 °C) for 1 h to ensure homogeneous dispersion.

BDMA triggers ring-opening reactions between the anhydride and epoxy groups, forming ester linkages and crosslinking the polymer network. This process occurs under nearly neutral conditions (pH ≈ 6.8) without the need for additional acid or base. The prepared mixture was then poured into a cylindrical mold (*ϕ* 25 mm × 50 mm) and thermally cured at 120 °C for 48 h in a constant-temperature oven. After curing, solidified semi-liquid gel samples with uniform morphology and adequate mechanical integrity were obtained for subsequent performance evaluation.

#### 4.2.2. Characterization

The liquid rubber plug was characterized using Fourier transform infrared spectroscopy (FTIR, Nicolet iS10, Thermo Fisher Scientific, Waltham, MA, USA) to investigate the epoxy modification effect. The microstructure of the fracture surface and adhesive interface was characterized using a scanning electron microscope (SEM, ZEISS Sigma 300, Carl Zeiss AG, Oberkochen, Germany). Before SEM observation, cured gel samples were cut into approximately 5 × 5 × 2 mm slices, followed by vacuum drying at 40 °C for 24 h to remove residual solvents. The dried samples were then sputter-coated with a thin layer of gold (≈5 nm) using an ion sputtering device to ensure conductivity and minimize charging during imaging. The thicknesses of the adhesive interface between the liquid rubber plug and the wall surface were characterized by atomic force microscopy (AFM, Dimension ICON, Bruker Corporation, Billerica, MA, USA) to analyze the interfacial adhesive performance. The particle size distribution of the uncured gel formulation was measured using a laser diffraction particle size analyzer (Mastersizer 3000, Malvern Instruments Ltd., Malvern, UK). Samples were diluted in isopropanol and subjected to ultrasonication to prevent agglomeration before measurement. All tests were conducted at 25 °C.

#### 4.2.3. Compressive Strength

Samples of the liquid rubber plugs were weighed and configured. Then, 25 g samples were added to *ϕ* 40 × 25 mm standard sample molds and cured at 120 °C. The standard sample was tested using a Universal Testing Machine (UTM) to obtain the compressive strength. The compression rate was set at 1 mm/min. The compressive strength (P) was calculated using Equation (1). The compressive strength was measured in triplicate. The reported value is an average, with a standard deviation within ±3.1 MPa.

(1) $$p=\frac{4F_{p}}{\pi \cdot d^{2}}$$

where *p* is the compressive strength (MPa), *F_p_* is the test pressure (N), and *d* is the diameter of the sample (mm).

#### 4.2.4. Determination of Curing Time

After the preparation of the liquid rubber plug reaction solution, the apparent viscosity changed slightly at 25 °C and rapidly increased as the cross-linking reaction was carried out at 120 °C. The configured samples were placed in sample bottles and then placed in an oil bath at 120 °C for curing and timed. The curing time was determined by visual code sending. The sample bottle was inverted, and the resin was assumed to be cured when no deformation of the resin surface was observed. Each test was repeated three times, and the gelation time was reported as an average value with a variation of ±0.5 h.

#### 4.2.5. Rheological Characterization Method

The rheology of the liquid rubber plug reaction solution was characterized using a DV-II (AMETEK Brookfield, Middleboro, MA, USA). Viscosity–time curves and shear rate viscosities under different temperature conditions were measured separately. After the preparation of the liquid rubber plug reaction solution, the apparent viscosity–time curves were measured from 120 °C to 140 °C. The apparent viscosities of the reaction solutions with different diluents were tested at 25, 50, and 100 °C.

#### 4.2.6. Interfacial Adhesive Strength Measurement

After preparation of the liquid rubber plugs reaction solution, 70 g samples were added to an *ϕ* 80 × 40 mm standard sandstone mold. Piston test samples of 50 mm thickness were cured and prepared at 120 °C (temperature fluctuation within ±1 °C). The samples were tested using a Universal Testing Machine (UTM) to obtain the load–time curve. The measurement rate was set at 1 mm/min.

The adhesive strength was calculated using Equation (2). Measurements were repeated three times, and the average values were reported with error bars representing ± standard deviation (typically ±0.15 MPa).

(2) $$p=\frac{F}{S_{c}}=\frac{10F_{s}}{\pi \cdot hD}$$

where *p* is the interfacial adhesive strength of the resin–steel interface (MPa), *F_S_* is the load force (kN), *S_c_* is the surface area of the cylinder (cm^2^), h is the height of the resin (cm), and *D* is the outer diameter of the resin (cm).

#### 4.2.7. Plugging Property Experiment

**Figure 18:**
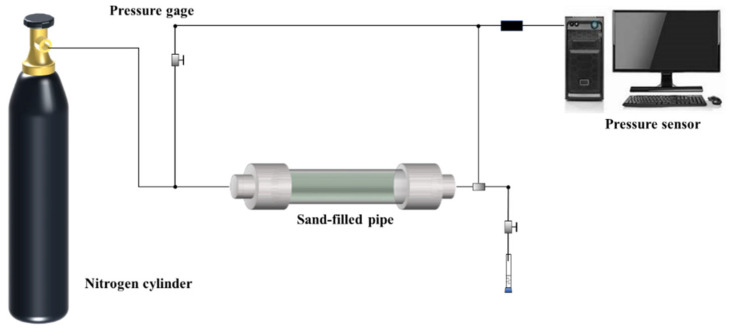
Simulation of casing leakage wellbore modules and displacement device.

The performance of the liquid rubber plugs in the plugging gas in the wellbores was simulated by using a sand-filled tube. The sand-filled tube was filled with quartz sand (100–120 mesh), and the physical properties of the packed bed were measured prior to the plugging tests. The porosity was determined to be approximately 27.8%, and the gas permeability was 132.6 mD under ambient conditions, as measured using a standard gas permeameter. First, 2 mL of weak gel was injected into a sand-packed tube *ϕ* 30 × 2.5 cm as pre-blocking to prevent the liquid rubber plugs from clogging the inlet of the sand-filled tube. Subsequently, a certain amount of the reaction solution for the liquid rubber plugs was added, and 2 mL of the gel solution was added. After the addition was completed, a pressure of 10 MPa was applied. The sand-filled tubes were placed in a constant-temperature oven at 120 °C for 24 h. As shown in Figure 18, the experimental apparatus was set up, and the gas displacement experiment was started.

To estimate the permeability reduction after gel plugging, the effective permeability *k* was calculated based on Darcy’s law using the breakthrough pressure gradient (∇P) under constant gas loading. Assuming a steady-state gas flow, a constant viscosity, and a linear flow regime, the permeability after plugging was calculated by

(3) $$k=\frac{Q\cdot \mu \cdot L}{A\cdot \nabla P}$$

where *Q* is the flow rate, *μ* is the gas viscosity (assumed constant), *L* is the sample length, *A* is the cross-sectional area, and ∇*P* is the measured pressure gradient. The initial permeability before plugging was measured to be 132.6 mD using dry nitrogen flow. The relative reduction in permeability was then calculated by comparing the effective post-plugging permeability with the initial value.

#### 4.2.8. Degradation Performance Test

Liquid rubber plug reaction liquids were placed in 50 mL sample bottles (m_0_), cured in a constant temperature oven at 120 °C, and conditioned for 24 h. The prepared degradation solution was placed in a sample bottle and aged at a constant temperature of 120~140 °C. The remaining mass (mi) of the liquid rubber plug was weighed, and the mass retention rate was calculated. The mass retention rate was calculated according to Equation (3).

(4) $$R=\frac{10-(m_{i}-m_{0})}{10}\times 100\%$$

where *R* is the mass retention rate, *m_i_* is the total mass of the sample bottle and liquid rubber plugs (g) and *m_0_* is the mass of the sample bottle (g).

## Figures and Tables

**Table 1:** Photographic comparison of cured liquid rubber plug samples before and after compression testing at different diluent dosages. The deformation modes evolve from minor cracks (8–10 wt%) to ductile bulging (14–16 wt%), indicating increased toughness and plastic resilience at higher diluent contents, consistent with the measured compressive strength trends.

Diluent Dosage	8	10	12	14	16
Initial state	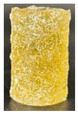	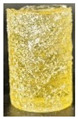	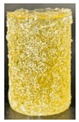	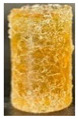	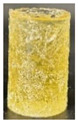
Repture state	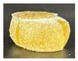	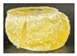	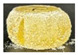	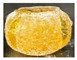	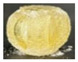

**Table 2:** Photographs of liquid rubber plug samples cured with varying OTS dosages, showing differences in interfacial morphology and adhesion quality at the tubing interface. The 0.25 wt% sample exhibits a distinct bonded polymer layer with roughened fracture edges, indicating strong interfacial anchoring. Excessive OTS (>0.5 wt%) results in irregular bonding, surface film delamination, and loss of cohesive continuity.

Diluent Dosage	0	0.25%	0.5%	0.75%	1%
Initial state	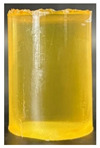	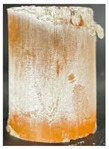	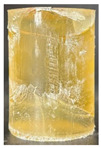	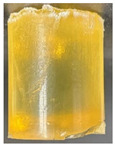	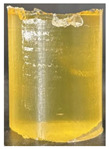

**Table 3:** Post-plugging permeability and reduction efficiency of the liquid rubber plug system at different injection volumes, calculated based on breakthrough pressure gradients using Darcy’s law.

Injection Volume (PV)	Breakthrough Pressure Gradient (MPa/m)	Calculated Post-Plugging Permeability (mD)	Permeability Reduction (%)
0.3 PV	19.11	19.04	85.6%
0.5 PV	24.50	24.51	81.5%
0.7 PV	34.38	34.32	74.1%
0.9 PV	50.59	50.47	61.9%

## Data Availability

The figures and tables used to support the findings of this study are included in the article.

## References

[1] Yang F., Li F., Ji R., Yu X., Yang H., Su G. (2024). Self-Degradable Rubber Plug for Temporary Plugging and Its Degradation Mechanism. Gels.

[2] Chen X., Lu X., Liu P., Du J., Liang C., Huang Q., Zhu D., Liu F. (2024). A critical review of key points in temporary plugging fracturing: Materials, injection, temporary plugging, and design. Geoenergy Sci. Eng..

[3] Anya A., Emadi H., Watson M. (2023). A novel apparatus and method for lab-scale study of wellbore integrity using CT imaging and analysis. J. Pet. Sci. Eng..

[4] Wang Y., Liu D., Liao R., Zhang G., Zhang M., Li X. (2022). Study of adhesive self-degrading gel for wellbore sealing. Colloids Surf. A Physicochem. Eng. Asp..

[5] Klishin S.V., Klishin V.I. (2020). Packer Sealing–Wellbore Interaction in Hydraulic Fracturing in Coal Seams. J. Min. Sci..

[6] Yang H., Lv Z., Li Z., Guo B., Zhao J., Xu Y., Xu W., Kang W. (2023). Laboratory evaluation of a controllable self-degradable temporary plugging agent in fractured reservoir. Phys. Fluids.

[7] Zhao X., Yang N., Liang H., Wei M., Ma B., Qiu D. (2024). The Wellbore Temperature and Pressure Behavior during the Flow Testing of Ultra-Deepwater Gas Wells. Fluid Dyn. Mater. Process..

[8] Bai Y., Pu W., Jin X., Shen C., Ren H. (2024). Review of the micro and Macro mechanisms of gel-based plugging agents for enhancing oil recovery of unconventional water flooding oil reservoirs. J. Mol. Liq..

[9] Huo J., Liu X., Zhao J., Zhang X., Zhang S., Wei C. (2025). Urea formaldehyde resin used as plugging agent in fractured and caved reservoirs: Preparation, optimization and modification. Polymer.

[10] Henriques C., Taleghani A.D. (2023). An evaluation of fiber-based lost circulation material for fracture plugging using simulations. Geoenergy Sci. Eng..

[11] Zhu D.-Y., Fang X.-Y., Sun R.-X., Xu Z.-H., Liu Y., Liu J.-Y. (2021). Development of degradable pre-formed particle gel (DPPG) as temporary plugging agent for petroleum drilling and production. Pet. Sci..

[12] Yuan C.-D., Pu W.-F., Jin F.-Y., Zhang Y.-C., Jia H., Zhao T.-H. (2014). Performance of Oil-Based Cement Slurry as a Selective Water-Plugging Agent in High-Temperature and High-Salinity Cave-Fractured Carbonate Reservoirs. Ind. Eng. Chem. Res..

[13] Jia H., Yang X.-Y., Zhao J.-Z. (2019). Development of a Novel In-Situ-Generated Foamed Gel as Temporary Plugging Agent Used for Well Workover: Affecting Factors and Working Performance. SPE J..

[14] Zhou H., Wu X., Song Z., Zheng B., Zhang K. (2022). A review on mechanism and adaptive materials of temporary plugging agent for chemical diverting fracturing. J. Pet. Sci. Eng..

[15] Lou Z., Wang K., Yao H., Zhao W., Qin H., Wu Z., Wei G. (2025). A novel dynamic filling material for plugging fractures around underground gas extraction boreholes: Experimental and engineering performances. Energy.

[16] Su X., Qi N., Han Z., Li X., Yan J., Chen S. (2023). Flow and plugging behavior of foams in fractures of fractured reservoirs. Colloids Surf. A Physicochem. Eng. Asp..

[17] Yang J.-B., Bai Y.-R., Sun J.-S., Lv K.-H. (2024). Curing kinetics and plugging mechanism of high strength curable resin plugging material. Pet. Sci..

[18] Yuan Z., Cao Z., Wu R., Xu Q., Xu H., Wu H., Jin B., Wu W., Zheng J., Wu J. (2023). Mechanically robust and rapidly degradable hydrogels for temporary water plugging in oilfields. J. Polym. Sci..

[19] Lai N., Tian Y., Wang J., Xu H., Tang L. (2024). Establishment of temperature-strength response prediction model of supramolecular gel temporary plugging agent by response surface method analysis. J. Appl. Polym. Sci..

[20] Leng G.-Y., Yan W., Ye H.-M., Yao E.-D., Duan J.-B., Xu Z.-X., Li K.-P., Zhang J.-R., Li Z. (2023). Evaluation of the injection and plugging ability of a novel epoxy resin in cement cracks. Pet. Sci..

[21] Chen Y., Li Y., Peng Y., Zhang D., Ye J., Jiang Y. (2024). Preparation of Low-Viscosity Epoxy Resin Sealing Agent and Evaluation of Injection, Plugging, and Degradation Properties. ACS Omega.

[22] Kuroyanagi M., Yamaguchi A., Hashimoto T., Urushisaki M., Sakaguchi T., Kawabe K. (2022). Novel degradable acetal-linkage-containing epoxy resins with high thermal stability: Synthesis and application in carbon fiber-reinforced plastics. Polym. J..

[23] Zhao X., Long Y., Xu S., Liu X., Chen L., Wang Y.-Z. (2023). Recovery of epoxy thermosets and their composites. Mater. Today.

[24] Rani M., Choudhary P., Krishnan V., Zafar S. (2021). A review on recycling and reuse methods for carbon fiber/glass fiber composites waste from wind turbine blades. Compos. Part B Eng..

[25] Torres-Herrador F., Eschenbacher A., Blondeau J., Magin T.E., Geem K.M.V. (2022). Study of the degradation of epoxy resins used in spacecraft components by thermogravimetry and fast pyrolysis. J. Anal. Appl. Pyrolysis.

[26] Sandro Dattilo G.C. (2022). Paolo Maria Riccobene, Concetto Puglisi, Lorena Saitta, Full Recycling and Re-Use of Bio-Based Epoxy Thermosets: Chemical and Thermomechanical Characterization of the Recycled Matrices. Polymers.

[27] Piñero-Hernanz R., Dodds C., Hyde J., García-Serna J., Poliakoff M., Lester E., Cocero M.J., Kingman S., Pickering S., Wong K.H. (2008). Chemical recycling of carbon fibre reinforced composites in nearcritical and supercritical water. Compos. Part A Appl. Sci. Manuf..

[28] Kim Y.N., Kim Y.-O., Kim S.Y., Park M., Yang B., Kim J., Jung Y.C. (2019). Application of supercritical water for green recycling of epoxy-based carbon fiber reinforced plastic. Compos. Sci. Technol..

[29] Yang P., Zhou Q., Yuan X., van Kasteren J., Wang Y. (2012). Highly efficient solvolysis of epoxy resin using poly(ethylene glycol)/NaOH systems. Polym. Degrad. Stab..

[30] Zhang N., Wu S., Wang C., Cui X., Zhao T., Yuan L., Qi Y., Hou X., Jin H., Deng T. (2022). Efficient catalytic degradation of anhydride-cured epoxy resin by amphiphilic molecule catalysts. Green Chem..

[31] Gao N., Lu Y., Li J., Zhao F., Ru M., Zhao S., Xiang S., Fu F., Diao H., Liu X. (2024). A fully degradable epoxy resin based on a nontoxic triphenol derived from diphenolic acid and eugenol. Polym. Chem..

[32] Wu Y., Hu Y., Lin H., Zhang X. (2024). An anhydride-cured degradable epoxy insulating material exhibiting recyclability, reusability, and excellent electrical performance. Green Chem..

[33] Liu X., Zhang Z., Luo Y., Sun J., Tian P., Liu H., Mao Z., Kan S., Yu X. (2024). Curing characteristics and performance of a bio-based polyurethane engineered sealant with high bonding strength: Effect of R value and chain extender content. Constr. Build. Mater..

[34] Zhang Z., Liu X., Ban X., Ji J., Tian P., Yang Y. (2025). The influence of filler types and content on the curing behavior and properties of a bio-based polyurethane engineered sealant. Int. J. Adhes. Adhes..

[35] Mane S., Ponrathnam S., Chavan N. (2015). Role of interfacial tension of solvating diluents and hydrophilic–hydrophobic cross-Linkers in hyper-cross-linked solid supports. Ind. Eng. Chem. Res..

[36] Nie W., Douglas J.F., Xia W. (2023). Competing effects of molecular additives and cross-link density on the segmental dynamics and mechanical properties of cross-linked polymers. ACS Eng. Au.

[37] He W., Wang Y., Li X., Ji Y., Yuan J., Yang W., Yan S., Yan J. (2024). Sealing the Pandora’s vase of pancreatic fistula through entrapping the digestive enzymes within a dextrorotary (D)-peptide hydrogel. Nat. Commun..

[38] Fang T., Ren F., Wang B., Hou J., Wiercigroch M. (2025). Multi-scale mechanics of submerged particle impact drilling. Int. J. Mech. Sci..

[39] Yu H., Zhao Z., Taleghani A.D., Lian Z., Zhang Q. (2024). Modeling thermal-induced wellhead growth through the lifecycle of a well. Geoenergy Sci. Eng..

